# Balancing the struggle to live with dementia: a systematic meta-synthesis of coping

**DOI:** 10.1186/s12877-019-1306-9

**Published:** 2019-10-30

**Authors:** Guro Hanevold Bjørkløf, Anne-Sofie Helvik, Tanja Louise Ibsen, Elisabeth Wiken Telenius, Ellen Karine Grov, Siren Eriksen

**Affiliations:** 1 Norwegian National Advisory Unit on Ageing and Health (Ageing and Health), Postbox 2136, N-3103 Tønsberg, Norway; 2 Department of Public Health and General Practice, Norwegian University of Science and Technology, Trondheim, Norway; 3 Department of Nursing and Health Promotion, Oslo Metropolitan University, Oslo, Norway; 4 VID Spesialized University, Faculty of Health Studies, Oslo, Norway

**Keywords:** Dementia, Meta-synthesis, Interviews, Coping, Life world perspective, Person’s experiences

## Abstract

**Background:**

People with dementia describe experiences of loss that threaten their autonomy and ability to contribute to society. They often have difficulties with orientation, loss of roll function, and fear about the future, and need help from others. An increasing body of literature also focuses on how people with dementia search for meaning and maintaining of quality to life, and how they find strategies to live with dementia. A review of the scientific literature on coping and dementia is warranted and can help to advice and inform healthcare personnel and decision makers on how they can support and plan for appropriate healthcare services for people with dementia. The aim of this systematic meta-synthesis was therefore to interpret and synthesize knowledge regarding people with dementia’s experience of coping.

**Methods:**

We conducted a systematic, computerised search of Medline, Embase, Cinahl Complete, PsycINFO and Age Line combining MeSH terms and text words for different types of *dementia* with different descriptions of *experience.* Studies comprised 1) a sample of people with dementia, 2) a qualitative interview as a research method and 3) a description of experiences of coping were included. The search resulted in 7129 articles, of which 163 were read in full text, 80 were excluded due to the exclusion criteria or low quality according. The analysis was conducted in line with qualitative content analyses.

**Results:**

This interpretative qualitative meta-synthesis included 74 articles of good quality encompassing interviews with 955 persons with dementia. The material revealed two main resources of coping: (1) Humour and (2) Practical and emotional support, and four overall strategies in which people with dementia cope with the challenges they experience: (1) Keep going and holding on to life as usual; (2) Adapting and adjusting to the demands from the situation; (3) Accepting the situation; and (4) Avoiding the situation A comprehensive understanding of the categories led to the latent theme: *Balancing the struggle of living with dementia*.

**Conclusion:**

This meta-synthesis indicates that people with dementia cope in different ways and using several parallel strategies. This insight is essential in dementia care to facilitate a supportive environment.

## Background

Dementia is a common term for a chronic functional decline caused by disease or damage in the brain, where Alzheimer’s disease is the most common [1, 2]. During progression of this decline the need for help from others are unavoidable and the focus moves from maintenance of daily functioning and activities in early phase towards comfort and well-being in late stage [3]. In the late, severe stage of dementia, the patients will be fully dependent on others and patients will eventually die [2, 4]. According to Alzheimer’s Disease International [5], the World Health Organization [6] and the Norwegian Dementia Plan 2020 [7], dementia treatment and care need to be based on the values of person-centered care [8]. Thus, in providing the appropriate treatment and support in accordance to people with dementia’s own needs and fundamental human rights, knowledge about how people with dementia experience and cope with their current and future life-situation is fundamental.

To live with dementia is an idiosyncratic experience as dementia influence each individual differently. Thus, a person becoming ill with a dementia will in different ways become dependent on own and surrounding resources for coping as the illness progresses. The concept ‘coping’ refers to the struggle to overcome and manage the stress from adapting in life [9]. Coping strategies are defined as “cognitive and behavioural efforts to master, reduce, or tolerate the internal and/ or external demands that are created by the stressful transaction” (p.843) [10]. Folkman & Lazarus’ transactional perspective on coping highlights people and surrounding environment to be in an ongoing reciprocal relationship. Life-stressors are constantly being evaluated in a process of appraisals where perceptions of available internal and external resources (primary appraisals) affect choice of strategies used to cope with the situation (secondary appraisal) [11]. The strategies involve approaches aiming to alter the stressful situation (i.e. problem-focused coping strategies), as well as regulation of emotional distress associated with the situation (i.e. emotion-focused coping strategies) [11, 12]. Thus, the strategies for coping depend on appraisals of the situational context and of personal factors; altogether constituting the individual’s available coping resources [11, 13]. Normally, people alternate between problem- and emotional-focused coping strategies, but in more severe and acute stress the use of all available strategies is triggered in a global coping response [11, 14–17]. A global coping response is defined as a response in acute and severe stress where you use: “combinations of almost all of the problem-focused and emotion-focused coping strategies, indicating the use of a substantial global coping response” ([17]p., 949).

During the last decade, a growing body of research has emerged that focus on how people experience and cope with dementia [18–20]. A review of the scientific literature on coping and dementia is therefore warranted and can help to advice and inform healthcare personnel, decision makers and informal carers on how they can support and plan for appropriate healthcare services for people with dementia.

## The review

### Design

An interpretative qualitative meta-synthesis was conducted [21, 22]. The review includes cross-sectional and longitudinal qualitative interview-studies describing coping in different ways.

## Methods

**Fig. 1:**
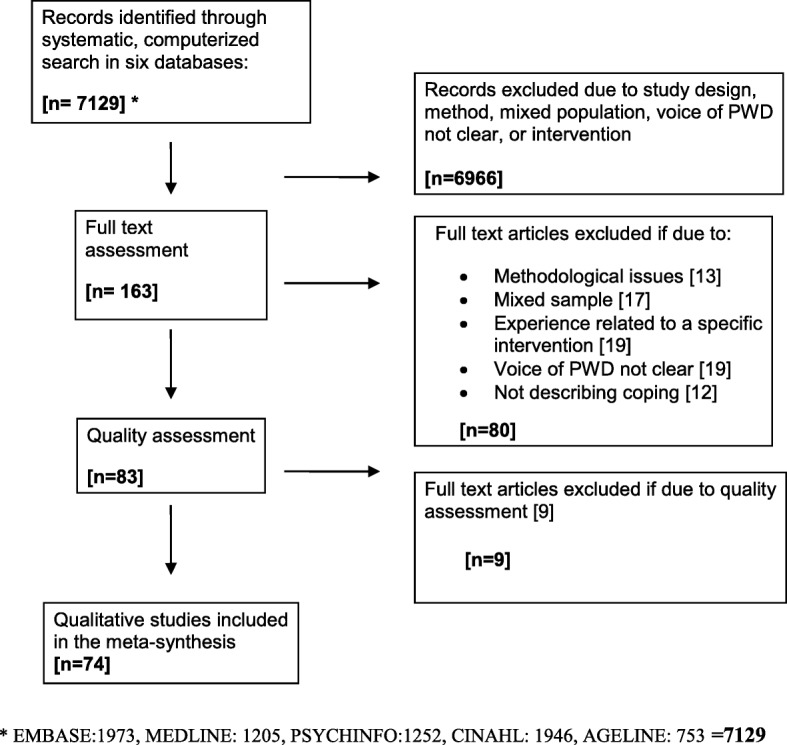
Flow chart, literature search

The methodology used for this meta-synthesis has also been used by some of the authors in previous published work [23, 24]. We conducted a systematic search combining different words for dementia and experience. The search was conducted in five databases: Age Line, Cinahl Complete, Embase, Medline and PsycINFO. See Table 1 for an overview of the search strategy as a whole. The search was restricted to peer-reviewed qualitative research studies in English language published between January 2004 and June 2018. We identified 7129 articles, of which 163 qualified for inclusion. Details of the inclusion og exclusion process is described in flowchart, Fig. 1.

**Table 1:** Search terms

Dementia	Experience
AGELINE Dement* OR Presenile dement* OR Senile dement* OR Alzheimer* OR Multi-infarct dement* OR Lewy Body dement* OR Vascular dement* OR Frontotemporal dement*] TX [all text] OR [dementia OR alzheimers disease OR alzheimers* OR lewy body*] SU [Subject]	AGELINE [Personal experience* OR Experience* OR Lived experience* OR Life experience* OR Patient experience* OR Subjective experience* OR First-person] TX AND [nursing methodologies OR case study OR constant comparison OR content analysis OR descriptive study OR discourse analysis OR ethnography OR exploratory OR feminist OR grounded theory OR hermeneutic OR interview OR narrative OR naturalistic OR participant observation OR phenomenology OR qualitative research OR qualitative methods OR qualitative study]
CINAHL [Dementia OR Dementia, presenile OR Dementia, senile OR Alzheimer’s disease OR Dementia, multi-infarct OR Lewy Body Disease OR Dementia, vascular] MESH OR [Dement* OR Presenile dement* OR Senile dement* OR Alzheimer* OR Multi-infarct dement* OR Lewy Body dement* OR Vascular dement* OR Frontotemporal dement*] TX [all text]	CINAHL [Life N1 Experience] OR [Personal experience* OR Experience* OR Lived experience* OR Life experience* OR Patient experience* OR Subjective experience* OR First-person] TX
EMBASE [Dementia OR Dementia, presenile OR Dementia, senile OR Alzheimer’s disease OR Dementia, multi-infarct OR Lewy Body Disease OR Dementia, vascular OR Dementia, frontotemporal] Keyword [KW] OR [Dement* OR Presenile dement* OR Senile dement* OR Alzheimer* OR Multi-infarct dement* OR Lewy Body dement* OR Vascular dement* OR Frontotemporal dement*] TW	EMBASE: [Personal experience* OR Experience* OR Lived experience* OR Life experience* OR Patient experience* OR Subjective experience* OR First-person] TW
MEDLINE [Dementia OR Dementia, presenile OR Dementia, senile OR Alzheimer’s disease OR Dementia, multi-infarct OR Lewy Body Disease OR Dementia, vascular] MESH OR [Dement* OR Presenile dement* OR Senile dement* OR Alzheimer* OR Multi-infarct dement* OR Lewy Body dement* OR Vascular dement* OR Frontotemporal dement*] TW	MEDLINE AND [Personal experience* OR Experience* OR Lived experience* OR Life experience* OR Patient experience* OR Subjective experience* OR First-person] TW
PSYCHINFO [Dementia OR Dementia, presenile OR Dementia, senile OR Alzheimer’s disease OR Dementia, multi-infarct OR Lewy Body Disease OR Dementia, vascular] MESH OR [Dement* OR Presenile dement* OR Senile dement* OR Alzheimer* OR Multi-infarct dement* OR Lewy Body dement* OR Vascular dement* OR Frontotemporal dement*] Tw	PSYCHINFOR [Life Experience] Mesh OR [Personal experience* OR Experience* OR Lived experience* OR Life experience* OR Patient experience* OR Subjective experience* OR First-person] TW

To minimize the risk of mistakes and ensure consistency in quality, the screening of articles was performed by pairs of authors, in line with PRISMA standard [25]. Disagreements was resolved by discussions in the whole group. Potential articles were read in full text and included if: (1) the sample consisted of people with dementia only; (2) the research method was qualitative interview; (3) the voice of people with dementia was clearly described; and (4) the article included a description of experiences of coping. Articles were excluded if: (1) dementia was described as probable or possible; (2) there was a mixed sample with people with dementia and people with other diagnoses, and (3) the paper described experience from a specific intervention.

### Assessment of the quality of the studies

Eighty-three of the articles were reviewed according to the CASP (Critical Appraisal Skills Programme) criteria for qualitative studies [26]. The quality of the studies was also assessed by pairs of authors. If disagreement that could not be resolved by discussion occurred, the group involved one of the other authors. We used the following nine criteria of the CASP: (1) a clear statement of aims; (2) appropriate choice of the method; (3) appropriate research design; (4) congruence between the recruitment strategy, aims and research; (5) the data collection method addresses the research issue; (6) a relationship between the researcher and the participant was considered; (7) ethical issues were considered; (8) the process of data analysis was sufficiently rigorous; and (9) a clear statement of the findings. Each criterion was given an equal weight (i.e., 1 point) for a maximum score of nine for each quality assessment per article. We considered a score of nine as high methodological quality and a score of 7–8 points was considered moderate quality (see Table 2). We included studies with a moderate or high quality. Nine studies were excluded due to low quality and scores below 7.

**Table 2:** Quality assessment of studies included

Study	Criteria^a^									Total /9	Quality
	1	2	3	4	5	6	7	8	9		
Aldridge H et al. 2017 [27]	+	+	+	+	+	–	+	+	+	8	Moderate
Aminzadeh F et al. 2009 [28]	+	+	+	+	+	–	+	+	+	8	Moderate
Aminzadeh F et al. 2010 [29]	+	+	+	+	+	–	–	+	+	7	Moderate
Atta-Konadu E et al. 2011 [30]	+	+	+	+	+	–	–	+	+	7	Moderate
Barrett C and Crameri P 2015 [31]	+	+	+	–	+−	–	+	+	+	7	Moderate
Beattie et al. 2004 [32]	+	+	+	+	+	–	+	+	+	8	Moderate
Borley G and Hardy S 2017 [33]	+	+	+	+	+	–	+	+	+	8	Moderate
Bronner K et al. 2016 [34]	+	+	+	+	+	–	–	+	+	7	Moderate
Brorsson et al. 2011 [35]	+	+	+	+	+	–	+	+	+	8	Moderate
Chaplin R et al. 2016 [36]	+	+	+	+	+	–	+	+	+	8	Moderate
Clare L et al. 2008 [37]	+	+	+	+	+	–	+	+	+	8	Moderate
Clemerson G et al. 2014 [38]	+	+	+	+	+	–	+	+	+	8	Moderate
Dalby P et al. 2012 [39]	+	+	+	+	+	+	+	+	+	9	High
Derksen E et al. 2006 [40]	+	+	+	+	+	–	+	+	+	8	Moderate
De Witt 2009 [41]	+	+	+	+	+	–	+	+	+	8	Moderate
De Witt 2010 [42]	+	+	+	+	+	–	+	+	+	8	Moderate
Digby 2011 [43]	+	+	+	+	+	+	+	–	+	8	Moderate
Digby 2012 [44]	+	+	+	+	+	+	+	+	+	9	High
Duggan et al. 2008 [45]	+	+	+	+	+	–	+	–	+	7	Moderate
Fleming R et al. 2015 [46]	+	+	+	+	+	+	+	+	+	9	High
Frazer SM et al. 2011 [47]	+	+	+	+	+	–	+	+	+	8	Moderate
Genoe MR et al. 2010 [48]	+	+	+	+	+	–	+	+	+	8	Moderate
Genoe MR et al. 2012 [49]	+	+	+	+	+	–	+	+	+	8	Moderate
Genoe MR and Dupuis SL 2014 [50]	+	+	+	+	+	+	+	+	+	9	High
Gill L et al. 2011 [51]	+	+	+	+	+	–	+	+	+	8	Moderate
Gilmour JA and Huntington AD 2005 [52]	+	+	+	+	+	–	+	+	+	8	Moderate
Goodman C et al. 2013 [53]	+	+	+	+	+	–	+	+	+	8	Moderate
Hain D et al. 2014 [54]	+	+	+	–	+	–	+	+	+	7	Moderate
Harman G and Clare L 2006 [55]	+	+	+	+	+	+	+	+	+	9	High
Harris PB 2011 [56]	+	+	+	+	+	–	+	+	+	8	Moderate
Hedman R et al. 2013 [57]	+	+	+	+	+	–	+	+	+	8	Moderate
Hedman R et al. 2016 [58]	+	+	+	+	+	+	+	+	+	9	High
Heggestad A et al. 2013 [59]	+	+	+	+	+	+	+	+	+	9	High
Hellström I et al. 2015 [60]	+	+	+	–	+	–	+	+	+	7	Moderate
Herron RV and Rosenberg MW 2017 [61]	+	+	+	–	+	–	+	+	+	7	Moderate
Hillmann A et al. 2018 [62]	+	+	+	–	+	–	+	+	+	7	Moderate
Hulko W 2009 [63]	+	+	+	+	+	–	+	+	+	8	Moderate
Johannessen A et al. 2011 [64]	+	+	+	+	+	–	+	+	+	8	Moderate
Johannessen A et al. 2014 [65]	+	+	+	+	+ −	–	+	+	+	8	Moderate
Karlsson E et al. 2014 [66]	+	+	+	+	+	–	+	+	+	8	Moderate
Keller HH et al. 2010 [67]	+	+	+	+	+	+	–	+	+	8	Moderate
Langdon SA et al. 2007 [68]	+	+	+	+	+	+	+	+	+	9	High
Lawrence RM et al. 2009 [69]	+	+	+	+	+	+	+	+	+	9	High
Lee SM et al. 2014 [70]	+	+	+	+	+	–	+	–	+	7	Moderate
Mac Kinley E 2009 [71]	+	+	+	+	+	–	+	+	+	8	Moderate
MacRae H 2011 [72]	+	+	+	+	+	+	+	+	+	9	High
Mazaheri M et al. 2013 [73]	+	+	+	+	+	+	+	+	+	9	High
Merrick R et al. 2016 [74]	+	+	+	+	+	–	–	+	+	7	Moderate
Mjørud M et al. 2017 [75]	+	+	+	+	+	–	+	+	+	8	Moderate
Molyneaux VJ et al. 2011 [76]	+	+	+	+	+	+	+	+	+	9	High
Mushi D et al. 2014 [77]	+	+	–	+	+	–	+	+	+	7	Moderate
Nowell ZC et al. 2013 [78]	+	+	+	+	+	+	+	+	+	9	High
Nygård L 2008 [79]	+	+	+	+	+	–	–	+	+	7	Moderate
Pesonen HM et al. 2013 [80]	+	+	+	+	+	–	+	+	+	8	Moderate
Phinney A 2011 [81]	+	+	+	+	+	–	+	+	+	8	Moderate
Pipon-Young FE et al. 2012 [82]	+	+	+	+	+	–	+	+	+	8	Moderate
Read ST et al. 2017 [83]	+	+	+	+	+	–	+	+	+	8	Moderate
Roach P et al. 2016 [84]	+	+	+	+	+	–	+	+	+	8	Moderate
Rostad D et al. 2013 [85]	+	+	+	+	+	–	+	+	+	8	Moderate
Sandberg L et al. 2017 [86]	+	+	+	+	+	–	+	+	+	8	Moderate
Sharp BK 2017 [19]	+	+	+	+	+	–	+	–	+	7	Moderate
Sixsmith A and Gibson G 2007 [87]	+	+	+	+	+	–	+	+	+	8	Moderate
Stephan A et al. 2018 [88]	+	+	+	+	+	–	+	+	+	8	Moderate
Svanström R et al. 2015 [89]	+	+	+	+	+	–	+	+	+	8	Moderate
Tak SH et al. 2015 [90]	+	+	+	+	+	–	+	+	+	8	Moderate
Tolhurst E and Weicht B 2017 [91]	+	+	+	+	+	+	+	+	+	9	High
Thein et al. 2011 [92]	+	+	+	+	+	–	+	–	+	7	Moderate
van Vliet D et al. 2017 [20]	+	+	+	+	+	–	+	+	+	8	Moderate
Van Zadelhoff E et al. 2011 [93]	+	+	+	+	+	–	+	+	+	8	Moderate
Vernooij-Dassen M et al. 2006 [94]	+	+	+	+	+	–	–	+	+	7	Moderate
Vikström S et al. 2008 [95]	+	+	+	+	+	–	–	+	+	7	Moderate
Weaks D et al. 2015 [96]	+	+	+	+	+	+	+	+	+	9	High
Wolverson EL et al. 2010 [97]	+	+	+	+	+	–	–	+	+	7	Moderate
Öman A and Nygård L 2005 [98]	+	+	+	+	+	–	–	+	+	7	Moderate

^a^ CASP criteria 1.Clear research statement, 2.Qualitative methodology, 3.Research question appropriate, 4.Recruitment strategy, 5.Data collection, 6.Relationship researcher – participants described adequately, 7.Ethical considerations, 8.Data analysis, 9.Clear statements of findings

We included a total of 74 articles interviewing 995 people with dementia in the meta-synthesis. The studies are presented in Table 3. Fifty-three of the articles emphasized on the participants’ ability to provide informed consent and verbally articulate their experiences, but only 21 described the participants’ stage of dementia or level of cognitive function. However, all studies provided information about participants’ housing situation and relocation to long-term care can serve as an indicator of dementia being in a moderate to severe stage. Among the included studies,13 studies interviewed participants living in nursing homes or other care facilities, 57 studies included participants living at home, and 4 studies had a mixed sample. Individual interviews constituted the main data and were described in 64 of the studies. Four studies were based on interviews with dyads or pairs consisting of person with dementia and health care personnel, and six studies were based on focus group interviews including people with dementia. Most studies conducted single interviews with the participants, but 17 studies were based on repeated interviews and of those, nine studies carried out interviews over a period of more than 2 months and can be characterized as longitudinal studies.

**Table 3:** Presentation of studies included

Authors & year	Aim	Participants^a^	Method
Aldridge H, Fisher P & Laidlaw K, 2017 [27]	To expand on this limited understanding and gain a deeper insight as to how shame is experienced and made sense of by people with early-stage dementia by exploring this topic directly with the people themselves.	*N* = 5 persons diagnosed with dementia. AD (3), Vascular (1), Mixed/vascular (2) Diagnosis of dementia 2–6 months prior to interview. Age: 74–90 years Women: 33% UK	Semi-structured interview in own home. Analysis of interviews was based upon the IPA procedure outlined by Smith) and Yardley.
Aminzadeh F, Dalziel WB, Molnar FJ & Garcia LJ, 2009 [28]	To explore the subjective meaning of relocation for persons with dementia moving into residential care.	*N* = 16 persons diagnosed with dementia living at home and planning to move to residential care within 2 months. Canada	Individual in-depth interviews. Field notes as supplementary data. Analyses were guided by the work of Corbin & Strauss.
Aminzadeh F, Dalziel WB, Molnar FJ & Garcia L, 2010 [29]	To examine the significance of home at the time of relocation to residential care from the perspective of persons with dementia.	N = 16 persons diagnosed with dementia living at home and planning to move to residential care within 2 months. Canada	Individual in-depth interviews. Field notes as supplementary data. Analyses were guided by the work of Corbin & Strauss.
Atta-Konadu E, Keller HH & Daly K, 2011 [30]	To provide a broader perspective and insight into the food–related role shift experiences of husbands and their wives with dementia by presenting the accounts of both spouses in the couple	N = 9 persons diagnosed with dementia living in their home and their spousal care partners. Age range: 58–86 years Women: 100% Living with partner: 100% Canada	Participants were interviewed yearly over a three-year period (the last year only 5 husbands and wives were still involved). First couples interviewed in dyads, and then individual interviews were accomplished 1 week to 1 month later. Data were analyzed using the constant comparative method described by Corbin & Strauss.
Barrett C & Crameri P, 2015 [31]	To outline the experiences and needs of lesbian, gay, bisexual and trans Australians living with dementia – and their partners	N = 9 persons living with dementia, partners (21) and service providers. Age range 47–79 years Australia	In-depth interviews mostly face to face (20). Data were analyzed using qualitative data analysis for applied policy research in line with Ritchie and Spencer.
Beattie A, Gavin D-W, Gilliard J & Means R, 2004 [32]	To demonstrate how interviews can be conducted with younger people with dementia.	*N* = 14 participants who had received a diagnosis of dementia and were using services. UK	Semi-structured, individual in-depth interviews Data were transcribed and subjected to comparative textual analysis guided by the principles of Strauss & Corbin
Borley G & Hardy S, 2017 [33]	*To explore the lived experience of becoming cared for and the impact his has on identity and sense of self of women with Alzheimer’s disease.*	*N* = 8 women with mild-to-moderate AD, living in own home Mean age: 78 years (range 74–83 years) Mean MMSE: 20 (range 15–26) scored within the last 6 months Living with partner: 100% Requiring assistance with I-ADL: 100% UK	Individual semi-structured interviews performed twice (within 4 weeks). The first interview enabled the women to talk about their life and experience related to their diagnosis of AD. The second interview allowed for further exploration and clarification with open-ended questions. Data were examined with interpretative phenomenological analysis in line with Smith, Flowers & Larkin.
Bronner K, Perneczky R, McCabe R, Kurz A & Harmann J, 2016 [34]	To identify medical and social topics which become relevant in the period following diagnosis of AD, for which a decision may eventually need to be made and which has implications for the life and wellbeing of the persons with AD	N = 5 persons with AD, relatives (6) and professionals (13). Germany	Semi-structured face-to-face interviews. Data were analyzed using content analysis in accord with Mayring.
Brorsson A, Øhman A, Lundberg S. & Nygård L, 2011 [35]	To illuminate experiences of accessibility in public space in people with AD, with particular focus on placed, situations and activities that they found to be important for daily life	*N* = 7 persons diagnosed with early AD, living in ordinary housing Sweden	Repeated in-depth interviews. All, except for one informant, were interviewed twice. Data were analyzed using open coding in accord with Corbin and Strauss.
Chaplin R & Davidson I, 2016 [36]	To focus specially on the experiences of people developing a dementia while still in employment in the UK	*N* = 5 persons with AD still being employed. MMSE: score range 25–28 Age range: 58–74 years Women: 20% UK	Individual semi-structured interviews on a single occasion. Data were analyzed using interpretative phenomenological analysis.
Clare L, Rowland J, Bruce E, Surr C & Downs M, 2008 [37]	To explore the subjective experience of living with dementia in residential care and to understand the psychological impact of being in this situation	*N* = 81 persons diagnosed with dementia living in residential care homes. UK	An existing dataset consisting of individual unstructured conversations with people with dementia from a study of well-being in residential care were used. The number of conversations recorded with each participant ranged from 1 to 8. The total dataset consisted of 304 transcripts. Interpretative phenomenological analysis as guiding design.
Clemerson G, Walsh S & Isaac C, 2014 [38]	To explore the individuals’ subjective experiences of young-onset dementia	N = 8 persons diagnosed with AD living at home. MMSE: score range 17–21 Age range: 35–60 years Women: 12.5% Living with someone (partner or others): 75.0% UK	Individual semi-structured interviews were performed. Data were analyzed using interpretative phenomenological analysis.
Dalby P, Sperlinger DJ & Boddington S, 2012 [39]	To understand the experience of spirituality in the context of living with dementia. In addition, a second aim was to understand the experience of dementia in the context of spiritual belief	*N* = 6 persons diagnosed with dementia living at home (75%), in assisted living (12.5%) or in nursing home (12.5%). Age range: from 70-ties to 90-ties Women: 83.3% Living with partner: 16.7% UK	Individual semi-structured interviews with participants were performed. Data were analyzed using interpretative phenomenological analysis.
Derksen E, Vernooij-Dassen M, Gillissen F & Scheltens P, 2006 [40]	To describe and appraise the experiences, beliefs, and fears regarding the diagnosis of dementia in both patients and carers	N = 18 persons diagnosed with dementia and their family carers. Mean MMSE score: 22 (range 15–30) Mean age: 71 years Women: 20% Living with partner 85%. The Netherlands	Individual semi-structured interview with patient and the carer were performed separately. Two interviews with participants; the first 2 weeks after the diagnostic disclosure and 10 weeks later. Data were analyzed using the constant comparative method in line with Corbin & Strauss.
De Witt L, Ploeg J & Black M, 2009 [41]	To understand the meaning of living alone for older people with dementia.	N = 8 women diagnosed with mild to moderate AD or related dementia living alone in the community Canada	Repeated face-to-face, open-ended interviews. All, except for two informants, were interviewed twice. Data were analyzed using three techniques data analysis in accord with van Manen.
De Witt L, Ploeg J & Black M, 2010 [42]	To understand the meaning of living alone from the perspective of older people with Alzheimer disease or a related dementia.	N = 8 women diagnosed with mild to moderate AD or related dementia living alone in the community. Canada	Repeated face-to-face, open-ended interviews. All, except for two informants, were interviewed twice. Data were analyzed using three techniques data analysis in accord with van Manen.
Digby R, Moss C & Bloomer MJ, 2011 [43]	To understand how older patients with mild to moderate dementia experienced the transfer from acute to subacute care and settling-in period.	N = 8 persons with dementia staying in a sub-acute facility Australia	In-depth semi-structured interviews using the communication techniques recommended by Young and Manthorp . Data were analyzed using content analysis in accord with Hsieh and Shannon.
Digby R & Bloomer MJ, 2012 [44]	To elicit the perspectives of current inpatients with dementia, and their family carers, about the environment/design features that they believe are necessary for people with dementia, and their family carers.	N = 7 persons with dementia staying in a sub-acute facility and carers (4) Australia	In-depth semi-structured interviews
Duggan S, Blackman T, Martyr A & Van Schaik P, 2008 [45]	To explore the use of outdoor environment and how dementia impacts on it.	*N* = 22 persons diagnosed with early to moderate AD or vascular dementia living in their own home, and carers (11 spouses/partners, 2 daughters, 1 carer/housekeeper) UK	Semi-structured individual interviews. Data were analyzed using NVivo and further in line with grounded theory.
Fleming R, Kelly F & Stillfried G, 2015 [46]	To identify the environmental features that are desirable in buildings used to provide care for people with dementia nearing the end of their lives	*N* = 2 persons with young onset dementia, family carers (10) and health care personnel (5). Australia	Mixed method. Three focus group interviews. In addition, a survey with experts in environmental design of care facilities for older people (21). Interview data were analyzed using management software NVivo 8.
Frazer SM, Oyebode JR & Cleary A, 2011 [47]	To explore how women who live alone with dementia see themselves and how they cope in their everyday lives	*N* = 8 persons diagnosed with dementia (AD = 5) living in their own home. UK	Individual, semi-structured interviews were performed. Data were analyzed using interpretative phenomenological approach.
Genoe MR, Dupuis SL, Keller HH, Martin LS, Cassolato C & Edward HG, 2010 [48]	To explore the experience and meaning of food and mealtimes for persons with dementia living in the community and their primary partners in care	*N* = 27 persons diagnosed with dementia (AD = 25) living in their own home together with their primary family caregivers (19 spousal relationships, 8 adult-child relationships). Majority were in early stage of dementia Age range: 56–88 years Women: 59.3% Living with someone: 100% Canada	Dyad interviews were followed by individual interviews within 2 weeks were conducted. Data were analyzed using grounded theory approach as described by Charmaz - the constant comparative method.
Genoe MR, Keller HH, Martin LS, Dupuis SL, Reimer H, Cassolato C & Edward G, 2012 [49]	To explore the meaning and experience of change surrounding mealtimes for persons with dementia living in the community and their primary partners in care	N = 27 persons diagnosed with dementia (AD = 25) living in their own home together with their primary family caregivers (19 spousal relationships, 8 adult-child relationships). Majority were in early stage of dementia Age range: 56–88 years Women: 59.3% Canada	Dyad interviews were followed by individual interviews within 2 weeks were conducted. Data were analyzed using grounded theory approach as described by Charmaz - the constant comparative method.
Genoe MR & Dupuis SL, 2014 [50]	To explore how persons with dementia think about and describe leisure in the context of their lives	*N* = 4 persons with early stage dementia living in their own home. Age range: 70–82 years Women: 50% Living with partner: 50% (both men) Canada	Individual interviews with each participant were accomplished. Data were also collected through participant observation and photo voice. Data were analyzed using van Manen’s phenomenological reflection.
Gill L, White L & Cameron ID, 2011 [51]	To understand how people with dementia receiving community care services in their own homes, perceive interaction in the context of their service experience	N = 22 persons diagnosed with dementia receiving community care services in their own home. Australia	Individual semi-structured interviews were performed. Data were analyzed using thematic- and constant comparison analyses.
Gilmour JA & Huntington A, 2005 [52]	To explore the experiences of living with memory loss	N = 9 persons diagnosed with dementia living at home. New Zealand	Individual, semi-structured interviews using open questions were used. To assist participants, questions were provided on beforehand and many participants wrote reminder notes prior to the interview. Thematic analyses were undertaken.
Goodman C, Amador S, Elmore N, Machen I & Mathie E, 2013 [53]	To explore how people with dementia discuss their priorities and preferences for end-of-life care, and how this might inform subsequent discussions with family and practitioners	N = 18 persons diagnosed with dementia living in residential care homes. UK	Individual, semi-structured interviews in the form of a ‘guided-conversation’ were conducted as a part of a longitudinal mixed method study. Thematic analyses were undertaken.
Hain D, Touhy TA, Compton Sparks D & Engstrom G, 2014 [54]	To explore the experience of living with dementia from multiple perspectives, namely, the individual, spouse, and dyad of the person and spouse	N = 6 persons diagnosed with AD and their spousal caregiver (6). Mean MMSE: 23.3 (20–25) Mean age: 79.3 (71–85) years Women: 16.7% Living with partner: 100% USA	Individual, semi-structured interviews conducted from multiple perspectives; the individual, spouse, and dyad of the person and spouse. Analyses were performed using the Giorgi’s descriptive phenomenological approach.
Harman G & Clare L, 2006 [55]	To explore the experience of living with dementia with focus on what makes activities meaningful for people with dementia	*N* = 17 persons diagnosed with dementia living in residential care homes, in addition their family caregivers (8), and staff (15). UK	Focus group design with a constructed question guide with residents, staff and relatives of the residents were performed. Mind map notes. Data were analysed using grounded theory approach with contents analysis.
Harris PB, 2011 [56]	To study factors of importance for maintaining and retention of friendship in early stage dementia	N = 8 persons diagnosed with dementia (AD = 7) living in their home. Early stage of dementia Mean age: 75 (59–85) years Women: 100% USA	Individual in-depth interviews were performed. Data were analysed using grounded theory approach in accord with Glaser and Strauss
Hedman R, Hansebo G, Ternestedt BM, Hellström I & Norberg A, 2013 [57]	To explore the use of Harré’s social constructionist theory of selfhood to describe how people with mild and moderate AD express their sense of self.	*N* = 12 persons diagnosed with AD living in their own home. Sweden	Individual, semi-structured interviews were performed. Data were analysed using phenomenological approach in accord with Harré’s theory of social constructionist.
Hedman R, Hansebo G, Ternestedt BM, Hellström I & Norberg A, 2016 [58]	To describe how five people with mild and moderate AD express their personal attributes and life histories	N = 5 persons diagnosed with mild to moderate AD. Age range: 59–78 years Women: 60% Living with partners: 80% Sweden	10 support group sessions during an 8 months period. Data were analyzed using an abductive approach in accord with McAdams and Graneheim and Lundman.
Heggestad A, Nortvedt P & Slettebø A, 2013 [59]	To investigate how life in Norwegian nursing homes may affect experiences of dignity among persons with dementia	N = 5 persons diagnosed with dementia living in nursing home. Norway	Individual interviews and observations field notes were used. Data were analysed using qualitative phenomenological and interpretative hermeneutical approach in accord with Kvale & Brinkman.
Hellström I, Eriksson H & Sandberg J, 2015 [60]	To describe how older women with dementia express the importance of their homes and chores in everyday life	N = 7 women diagnosed with dementia. Age: 65–84 years Living with spouse: 100% Sweden	Supplementary secondary analysis of a longitudinal study exploring ways in which people with dementia and their spouses (20 couples) experienced dementia over time. Several individual interviews (3–5) were performed. Data were analyzed in accord with the method of qualitative description according to Sandelowski.
Herron RV & Rosenberg MW, 2017 [61]	To examine how people with dementia relate to and within their communities as well as their perceptions of community support service.	*N* = 46 community-dwelling people with dementia and their partners; spouse (39), daughter (2), sister (1), son (1). Diagnosis of dementia: 3 years (average) Age: 56–93 years Women: 43% Canada	Qualitative case-study approach. Semi-structured interviews. All but two participants had a partner in care present with them. NVivo-coding according to Charmaz
Hillmann A, Jones IR, Quinn C, Nelis SM & Clare L, 2018 [62]	(1) To identify the kinds of representations of dementia present in the accounts of those who speak for people with dementia. (2) To situate these stories within their wider social and cultural contexts, to ascertain the extent to which they reflect, contribute to or challenge existing representations of dementia. (3) To utilise Burchardt’s work (2016) to consider what the implications might be of their circulation and accumulation in a narrative economy of dementia.	N = 5 people living with dementia and their partners (4) AD (2), Vascular dementia (2), mixed (1). Age: 49–83 years Women: 40% UK	The participants were interviewed twice, a few months apart. Analyzed with constant comparative method in line with Glaser & Strauss and Silverman
Hulko W, 2009 [63]	To explore the experience of older people with dementia and in which way socio-culture plays a role in diverse dementia patients’ daily living	N = 8 persons diagnosed with dementia (AD = 7) living in their home and their relatives (50). Canada	Series of individual in-home interviews over 1–2 month and observation sessions were used. Data were analyzed in accord with grounded theory.
Johannessen A & Möller A, 2011 [64]	To find out how people experience living with early-onset dementia, and to assess the implications for practice and the development of further services	N = 20 young persons with a diagnosis of dementia. Norway	Individual, thematic interviews were conducted. Data were analysed in line with grounded theory according to Glaser and Strauss,
Johannessen A, Möller A, Haugen PK & Biong S, 2014 [65]	To investigate and interpret metaphorical expressions of the lived experiences of everyday life in people with young-onset dementia	*N* = 20 young persons with a diagnosis of dementia. Age: 54–67 years Women: 40% Living with spouse: 75% Norway	Individual, thematic interviews were conducted. Secondary analysis of the data in line with cognitive-semantic theory according to Lakoff & Johnson.
Karlsson E, Sävenstedt S, Axelsson K & Zingmark K, 2014 [66]	To explore how people with AD present their life story	N = 9 participants diagnosed with AD, living in their homes. MMSE: 19–25 Age: 60–81 years Women: 55.5% Living with spouse: 88.9% (8) Sweden	Individual, narrative interviews were conducted. Data were analyzed with the method for analysis of narrative in accord with Polkinghorne.
Keller HH, Martin LS, Dupuis S, Genoe R, Edward HG & Cassolato C, 2010 [67]	To explore the mealtimes to provide opportunity for social activity and emotional connection	N = 27 participants with early to mild stage of dementia living in their home and their next of kin (28). Canada	Active interviews with both individual and dyads were performed. Data were analyzed using grounded theory methodology in accord with Charmaz and team analysis.
Langdon SA, Eagle A & Warner J, 2007 [68]	To explore the social effects of diagnosis of dementia	N = 12 persons diagnosed with dementia living in their own home. MMSE range: 19–30 Mean age: 79 (range: 66–87) years Women: 50% UK	Individual semi-structured in-depth interviews were performed. Data were analyzed in accord with interpretative phenomenological approach.
Lawrence RM, Samsi K, Banerjee S, Morgan C & Murray J, 2011 [69]	The subjective reality of living with dementia from the perspective of three minority ethnic groups. Thoughts and other reactions to the diagnosis dementia	*N* = 30 persons diagnosed with dementia living at home or in sheltered accommodations (4). UK	Individual in-depth interviews were performed. Data were analyzed using grounded theory approach in accord with Glaser.
Lee SM, Roen K &Thornton A, 2014 [70]	To explore personal experiences of receiving a diagnosis and to investigate aspects of the experience of adjusting and adapting to dementia	*N* = 10 persons diagnosed with mild AD, living at home. Mean MMSE: 25 (22–30) Mean age: 69 (57–84) years Women: 70% Living with partner: 50% UK	Individual, semi-structured interviews were performed. Data were analyzed using interpretative phenomenological approach.
Mac Kinlay E, 2009 [71]	To examine spirituality and meaning in the experience of dementia of older Latvians who had immigrated to Australia during the war II	N = 3 persons diagnosed with dementia living in an aged-care facility. MMSE: 18–20 Age: 87–94 years Australia	Individual in-depth interviews were performed. Data were analyzed using grounded theory in accord with Strauss and Corbin.
MacRae H, 2011 [72]	To examine how others’ reactions to and treatment of persons living with early stage AD influence their experience of dementia	N = 9 persons diagnosed with early stage AD. living at home (7), in senior’s residence (1) or in a convent (1). Mean age: 74 years Women: 22.2% Living with partner: 44.4% Canada	Individual in-depth, interviews using a symbolic interactionist perspective were performed. Data were analysed using inductive emergent process in accord with Coffey & Atkinson, Lofland & Lofland, and Taylor & Bogdan.
Mazaheri M, Eriksson LE, Heikkilä K, NasraBadi AN, Ekman SL & Sunvisson H, 2013 [73]	To describe experience of living with dementia in Iran	*N* = 15 persons diagnosed with moderate AD, or Vascular dementia, living at home. Mean MMSE: 16.5 (range: 14–19) Mean age: 72 (range 60–87) years Women: 40% Living with someone: 80% Iran	Individual semi-structured interviews were performed. Data were analysed using content analysis in accord with Graneheim and Lundman.
Merrick K, Camic PM & O’Shaughnessy M, 2016 [74]	To enrich understanding of the experience of dementia from a relational perspective	N = 7 persons with dementia and their care partners (7). AD (4), frontotemporal dementia (1), vascular dementia (1) mixed (1). Age range: 65–87 years Women: 29% UK	Semi-structured dyad interviews. Data were analyzed using an interpretative phenomenological approach.
Mjørud M, Engedal K, Røsvik J & Kirkevold M, 2017 [75]	To investigate the personal experience of living in a nursing home over time and what makes life better or worse from the perspective of the person with dementia	N = 12 persons with dementia living in nursing home care units for persons with dementia Norway	Repeated individual, unstructured interviews 3 months apart. Field observations. Data were analyzed using phenomenological-hermeneutical analysis in accordance with Lindseth and Norberg.
Molyneaux VJ, Butchard S, Simpson J & Murray Cl, 2011 [76]	To understand ‘couple-hood’ as it is co-constructed by the couple when one partner has dementia.	N = 5 persons diagnosed with AD. and their partner living at home. UK	The couples were interviewed simultaneously. Data were analyzed using constructivist grounded theory approach in accord with Charmaz.
Mushi D, Rongai A, Paddick SM, Dotchin C, Mtuya C & Walker R, 2014 [77]	To explore the socio-cultural beliefs surrounding dementia and the life experience of people with dementia and their caregivers in the Tanzania	*N* = 41 persons diagnosed with dementia living at home and their caregivers, but only 25 persons with dementia were interviewed. Tanzania	Semi structured paired interviews (25) and individual interviews (16) with the caregiver alone were performed. Data were analyzed using content analysis.
Nowell ZC, Thornton A & Simpson J, 2013 [78]	To understand personhood by exploring the subjective experiences of those with dementia in UK	N = 7 people diagnosed with dementia living in dementia care units. UK	Individual semi-structured individual interviews were performed. Data were analyzed using an interpretative phenomenological approach.
Nygård L, 2008 [79]	To explore how people with dementia who live alone experienced the meaning of their everyday technology, such as telephone and electronic equipment, and the use of it.	N = 8 persons diagnosed with dementia living at home. Sweden	Repeated individual interviews and observations (for 3 weeks) were performed. Two to four sessions of interviews and observations pr. person, each session lasting between 1 to 2 h. Data were analyzed using a phenomenological, hermeneutical approach.
Pesonen HM, Remes AM & Isola A, 2013 [80]	To explore the shared experience of dementia from the viewpoint of people with newly diagnosed dementia and their family members, and to understand how they manage their lives after the diagnosis	N = 8 persons diagnosed with dementia (AD =6) living in their home or nursing home/assisted living facility (4) and their family members (8). Finland	Conversational, low structured face-to-face interviews. Unstructured observations were conducted during the interviews; field notes were written after each interview. Descriptive analysis using grounded-theory framework and constant comparative analysis in accord with Corbin & Strauss.
Phinney A, 2011 [81]	To understand how people with dementia understand their lives as making sense and worth living.	N = 9 persons with mild to moderate AD, living in own home. Canada	Repeated in-depth conversational interviews. Participant observation.
Pipon-Young FE, Lee KM, Jones F & Guss R, 2012 [82]	To explore the experiences of younger persons with dementia and *develop an understanding of helpful support* *To identify areas of the service in need for change*	N = 8 persons diagnosed with dementia living in their home. UK	Action research across three phases; semi-structured individual interviews and field notes were used. Data were analyzed using action research; interpretative approach including thematic analysis techniques in line with Charmaz and concept mapping in accord with McNiff & Whitehead.
Read ST, Toye C & Wynaden D, 2017 [83]	To explore the person with dementia’s expectations of their support needs and how they wish to live their lives	*N* = 24 persons diagnosed with dementia, living in own home. AD (8), Vascular dementia (3), Frontal Lobe dementias (3), Semanticdementia (1) and Posterior cortical atrophy (1). Eight partici-pants did not know the type of dementia) Women: 50% Living with someone: 70% Australia	Individual semi-structured interviews. Field notes and memos were also collected to add contextual meaning to data collected from participants. Questions focused on participants’ experiences of the onset of their dementia, the impact of the diagnosis on themselves and their family, plus their future expectations of living with dementia. This research used an application of the Grounded Theory (GT) method developed by Glaser and Strauss (2012).
Roach P, Drummond N & Keady J, 2016 [84]	To develop deeper understanding of the family experience of transition in early-onset dementia and to develop a representative model of this experience	*N* = 9 persons with early onset dementia and their family members (11) AD (7), mixed (1), posterior cortical atrophy (1) Age range: 58–68 years Women: 0 Canada	Individual, initial and follow-up semi-structured interviews. Data were analyzed in accord with a framework approach to qualitative data analysis by Ritchie and Spencer.
Rostad D, Hellzen O & Enmarker I, 2013 [85]	*To gain understanding of the lived experience of younger persons with dementia (< 65 years) who lived at home and suffered with early onset, and the meaning that could be found in their experiences.*	N = 4 persons diagnosed with dementia living in their home. Norway	Individual, narrative individual interviews in a conversational style with broad open-ended questions were used. Phenomenological hermeneutic approach to the analysis in line with Lindseth and Nordberg.
Sandberg L, Rosenberg L, Sandman P-O & Borell L, 2017 [86]	*T*o explore and better understand how people with dementia, living at home, experience risks in their daily life and how they handle these situations.	N = 12 persons with mild-to-moderate dementia, living in wn home AD (9), Vascular dementia (2), Levy Body (1) Mean age: 77 years (range 67–87) Women 50% Living with someone: 66.6% Sweden	Semi-structured individual interviews with open-ended questions The interviews were analysed in steps using a qualitative content analysis approach in line with Granheim & Lundman.
Sharp BK, 2017 [19]	To describes how people with dementia perceive their experiences of stress, and how people with dementia cope with the stress they experience?	*N* = 21 persons with dementia living in own home AD (13), Vascular dementia (7),Mixed (1) Mean age: 65.9 Women: 52.3% Living with someone:66.7% UK	Focus groups. The study is an interpretative phenomenological analysis (IPA) in accordance with Smith, Flowers, & Larkin. NVivo Qualitative Data Analysis program was used.
Sixsmith A & Gibson G, 2007 [87]	*To study: the role and importance of music in the lives and activities of the participants, the benefits they derived from music and music-related activities*	*N* = 26 persons diagnosed with dementia living in their home (16) or staying in care homes (10) and their family caregivers. Age: 62–96 years Women: 69.2% Living with someone: 70% UK	Individual interviews in their natural setting, at home (16–18) and in the care homes (8–10) were performed. Open ended interviews, which were loosely structured. Observational data from private home settings were gathered.
Stephan A, Bieber A, Hopper L, Joyce R, Irving K. Zanetti O, Portolani E, Kerpershoek L, Verhey F, de Vugt M, Wolfs C, Eriksen S, Røsvik S, Marques MJ, Gonçalves-Pereira M, Sjölund BM, Jelley H, Woods B & Meyer G, 2018 [88]	Explore the perspectives of people with dementia, their informal carers and health and social care professionals of accessing and using formal care and services. Aiming to improve the understanding of the facilitators and barriers to the access to and the use of formal dementia care for the further development of appropriate services and interventions.	*N* = 51 persons with dementia, 96 informal carers and 114 professionals All types of dementia included. Mean age: 76 (range 54–96) Living in own home: 92.2% Women: 54.9% Living with someone:60.8% Germany, Italy, Portugal, Sweden, Norway, UK, Ireland, the Netherlands	Focus groups. Qualitative content analysis using open coding was performed in each county, according to Elo & Kyngas H and Graneheim & Lundman. To ensure consistency and methodological rigor, a manual was provided to all the partners. The analysis was supported by the software MAXQD Aplus version 11 (VERBI GmbH, Berlin, Germany).
Svanström R & Sundler AJ, 2015 [89]	*To elucidate the phenomenon of living alone with dementia and having a manifest care need*	N = 6 persons with dementia lining in own homes. Sweden	Several conversational interviews and field notes. 32 visits with six participants. Data were analyzed in accord with an in-depth phenomenological analysis.
Tak SH, Kedia S, Tongumpun TM & Hong SE, 2015 [90]	*To describe types of current activity involvement and barriers to activities reported by nursing home residents with dementia*	*N* = 37 nursing home residents with dementia. USA	Individual short, open-ended interviews (31) and individual in-depth interviews (6) were performed. Data were analyzed in accord with descriptive, content analysis within ethnographic framework.
Tolhurst E & Weicht B, 2017 [91]	Explore how men with dementia seek to preserve their own personhood in response to the impacts of the condition. Explore how men with dementia seek to preserve their own personhood in response to the impacts of the condition. The authors claim that there is a lack of a masculine-gendered portrayal of the experience of dementia.	N = 14 men with dementia and their spouse, living at home Mild to moderate AD (12), Mild Levy Body (1), Moderate Vascular dementia (1) Mean age: 73.5 (range 58–89) UK	Two semi-structured dyad interviews of all 14 couples, with 6 months between each interview. Narrative analysis according to Riessman, 2008
Thein NW, D’ Souza G & Sheehan B, 2011 [92]	*To explore the subjective experience of people with dementia of the move to a care home.*	N = 18 persons with mild to moderate dementia moving in to nursing home UK	Repeated semi-structured individual interviews before and after moving to nursing home. Systematically coding with NVivo using the headings for the interview as major codes.
van Vliet D, Persoon A, Bielderman A, Bakker C, Koopmans RTCM & Gerritsen DL, 2017 [20]	Explore how people with YOD shape their daily lives to retain a sense of usefulness.	*N* = 18 persons with dementia living in own home, and 21 informal care givers AD (8), Fronto temporal (3) Vascular dementia (1), Mixed (1) Not specified (*n* = 5) Mean age: 63.5 (range 57–70) Women: 38.9% Living with someone: 75% The Netherlands	Focus groups, using a discussion guide with open-ended questions. Qualitative content analysis in line with Graneheim & Lundman and Elo & Kyngas. The analysis was supported by the softwear Atlas.ti.
Van Zadelhoff E, Verbeek H, Widdershoven G, van Rossum E & Abma T, 2011 [93]	*To investigate experiences of residents with dementia, their family and nursing staff* in group living homes for older people with dementia and their perception of the care process	N = 5 persons diagnosed with dementia living in a non-profit nursing home, in addition, residents’ family members (4) and staff (5). The Netherlands	Individual in-depth interviews with open-ended questions were performed separately with each of the participants. Observations and field notes were taken. Inductive and theoretical analysis was used.
Vernooij-Dassen M, Derksen E, Scheltens P & Moniz-Cook E, 2006 [94]	*To* prospectively describe and understand the impact of receiving a diagnosis for individuals and their family carers over time, in order to suggest best practice for services and practitioners	N = 18 persons with dementia living in their own home and their family carers. Mean MMSE: 22 (range 15–30) Mean age 71 years Women: 22.2% Living with someone: 83.3% The Netherlands	Individual semi structured interviews about 2 weeks and 12 weeks after diagnosis were performed of PWD and family caregivers. Constant comparative analysis using grounded theory in accord with Corbin & Strauss.
Vikström S, Josephson S, Stigsdotter-Neely A & Nygård L, 2008 [95]	*To* identify and describe how persons with dementia and their caregiving spouses perceive their own, their spouses’ and their mutual engagements in everyday activities.	N = 26 persons with dementia living in their home and their caregiving spouses (26). Sweden	Individual semi-structured individual interviews with open-ended questions were performed for PWD and caring spouse. Analyzed using constant comparative method in line with grounded theory by Corbin & Strauss.
Weaks D, Wilkinson H & McLeod J, 2015 [96]	*To explore the ways in which people with dementia, and those close to them, negotiated the task of disclosure of the diagnosis*	N = 5 persons with early AD living in their home, and persons close to them (18). Age: 68–79 years Women: 60% Living with partner: 80% UK	Sequential interviews combined with participant observation over a 6 months period. Data were analyzed with Grounded Theory approach in accord with Corbin and Strauss. NVivo Qualitative Data Analysis program was used.
Wolverson EL, Clarke C & Moniz-Cook E, 2010 [97]	*To investigate the subjective experience of hope of people with dementia*	N = 10 persons diagnosed with AD living in their home. UK	Individual semi-structured interviews with open-ended questions were performed. Data were analyzed using interpretative phenomenological approach in line with Smith.
Öhman A & Nygård L, 2005 [98]	To uncover and describe the meaning and motives for engagement in self-chosen daily life occupation for elderly individuals with AD dwelling in community.	N = 6 community-dwelling persons diagnosed with AD. Sweden	Repeated individual interviews and observations. Totally two or three times per person. A qualitative comparative analysis method was used in accord with Bogdan & Biklen.

^a^Sociodemographic information is described for those studies presenting such information

### Data abstraction and synthesis

The abstraction and synthesis were conducted according to the principles of the interpretative synthesis [99]. We focused on the development of concepts based on the data from primary studies and further developed and specified theories that integrated the concepts [100]. The analysis built on the principles of qualitative content analyses [101] and comprised six steps: In the *first step*, pairs of authors (TLI, EKG, EWT and SE) read all the 74 papers. Results from the papers describing coping, were extracted as direct citations into “meaning units” (TLI and SE); a form created for further analysis [101]. In the *second step*, two of the authors (TLI and SE) condensed the meaning units. This is a process were the content of the direct citation is being extracted using the meaning unit’s own language. In the *third step*, two other authors (GHB and ASH) labelled the condensed meaning units with codes in order to organize the material. In the *fourth step* all authors compared codes and identified similarities and differences in order to structure and gathering the codes into subcategories. In the *fifth step*, the eight subcategories identified through this process were ultimately gathered into four categories with subcategories presenting the manifest meaning of the material. Finally, in the *sixth step,* the *comprehensive understanding phase,* we summarized and reflected upon the results consisting of resources and strategies, in order to reach a presentation of the text as one overall latent theme [101].

## Results

The 74 articles included in this meta-synthesis described challenges and sources of stress that people with dementia encounters. The studies reported experience of loss of autonomy, control and connection. The participants see themselves as “different” and no longer normal [32]. The memory problems, other symptoms of dementia and the unpredictable progression of the disease lead to worry and anxiety. The participants experience that their needs change; the need to be looked after and to be taken care of increases, and furthermore, new social and emotional needs arise [36]. The studies describe the experience of being stigmatized, feeling embarrassed or stupid. Dementia threatens the identity and sense of worth and changes their roles and the relationship to others [30, 50]. The participants state that taking part in social events and meaningful activities become more difficult. Consequently, some feel that they lack competency and may not be contributing as much as they would like, for instance in household and society. Loss of social contact and meaningful activities lead to loneliness, isolation, emptiness or boredom and many people experience that it is necessary to adjust life expectations [50, 89]. The decline in function and abilities have great impact on daily life and some express that life loses its purpose and that happiness is gone. The participants describe concerns for the future [82]. The progression of dementia, and thereby the future, is unpredictable.

### Coping with dementia

The experience of challenges and stress forms the backdrop for the experience of coping and the coping strategies people with dementia use. It is interesting to note that only two of the 74 included studies explicitly aimed to explore coping [18, 47].

The material emphasized two essential resources of coping that goes across the entire material: *humour* and *social and emotional support*. *Humour* is a personal resource that can be used actively to handle the symptoms of dementia. By laughing instead of crying, humour can be used as a safeguard in overwhelming situations. An ability to see the funny side can reduce stress, distract negative mood and elicit positive emotions. *Social and emotional support* are external resources of coping and describes the backing and practical help received from others; family, friends and other relations. Social support is important for coping with rising demands in life with dementia (See Table 4).

**Table 4:** Results coping resources: Examples of condensed meaning units

Category	Humour [27, 40, 47, 49, 50, 63, 68, 73, 74]	Social and emotional support [30, 34, 40, 49, 51, 57, 66, 67, 72, 81, 94, 95]
Condensed meaning unit	Humour to cope with psychic pain [47]. Taking control of dementia by minimising its impact and using humour when describing coping strategies [47]. Using humour to cope with the painful awareness of memory decline [47]. Humour and spirituality to cope [49]. Using humour and laughing at one selves helped handle the change and challenge [50]. Using humour to help dealing with dementia [68]. Using humour as strategy [73]. Dismissing the significance of memory loss, and use humour to cope [63]. Using humour as a saviour [74]. Laughter and humour [27].	Taking part in activities leading to connection with others [66] Mobilizing resources by accessing external services and friends and families [49] Acknowledging the value of receiving support [51]. Holding on to their roles in valuable relationships [40]. Promoting reciprocity and maintenance of relationship through food role changes [30]. Sharing emotions [40]. Letting the family steer, represents a shifting orientation toward what is good and meaningful in life [81] Feeling lucky to be supported by family and home care [57]. Expects their relatives to take care of them. Postponing the decisions about the future [34]. Becoming stupid if you don’t talk to people [67] Fortunate to be married and being looked after [30]. Holding on to roles in social relationships [94]. Continuing to interact regularly with friends [72]. Sharing the diagnosis with their children [40]. Seeking information about dementia and support [40]. Nearness to the caregiver as an asset [95]

The analysis revealed four overall categories of coping strategies: (1) Keep going and holding on to life as usual; (2) Adapting and adjusting to the demands from the situation; (3) Accepting the situation; and (4) Avoiding the situation (See Table 5):

**Table 5:** Results coping strategies: Overview of categories and subcategories with examples of condensed meaning units

Category	Keep going and holding on to life as usual [19, 20, 27–29, 33–35, 37–41, 43, 47–50, 52, 54, 57, 58, 61, 62, 64–66, 68, 70–76, 78, 80–82, 85, 90, 91, 93, 94, 97, 98]	Adapting and adjusting to the demands from the situation [19, 20, 27, 28, 30, 32, 35–41, 43–50, 52–57, 59–64, 68, 70, 72–75, 77–83, 85–91, 93, 94, 96–98]	Accepting the situation [20, 33, 36–38, 41, 43, 47, 48, 50, 51, 53, 56, 57, 62, 64, 68–70, 72, 74, 75, 80–82, 85, 91, 92, 97]	Avoiding strategies [27, 31, 38, 39, 42, 43, 47, 49, 57, 71, 72, 86, 89, 92, 94]
Sub-categories	- *Preserving identity* - *Normalising the situation* - *Contributing to society*	- *Taking control and compensating* - *Reframing identity.*	- *Position in life*	-
Condensed meaning units	*Preserving identity* Living life according to one’s core values [47]. Good memories of childhood and adult life confirms one’s position/identity [37]. Holding on to the existing self-concept [38]. Remembering and reminding herself of who she was, her strengths and characteristics [39]. Stories and memories from the past helped preserve the person they felt themselves to be [39]. Holding on to their roles in valuable relationships [40]. Holding on to identity makes it able to sustain and support a restricted self [47]. Narrating memories of past achievement as positive aspects of life [57]. Previous occupations were a manifest as examples of competence [98]. Using happy memories for comfort in the present situation [75]. Drawing on past roles and status as a reminder of who they were in the present, despite their new and “strange territory” [78]. Asserting strength, they once had, to manage this period of their life [78]. Making reference to working roles that were undertaken in the past [91]. Identifying personality and coping styles to meet the future [28]. Relating stories of taking initiative to preserve their faith and control their lives [85]. Having survived an aggressive cancer makes dementia “just another setback” [91]. *Normalising the situation* Carry on as normal. Maintaining normality [34]. Keep telling myself that none of this is important [39]. Using a lot of energy to maintain a normal situation in daily life and preserving hope and willpower [85]. Not telling anybody about dementia, hoping to be seen as before [82]. Making extra efforts to behave according to the norms to avoid problems [73]. Normalization of memory loss decrease worry [50]. Normalizing the experience of dementia by comparing one-selves to others [70]. Explaining memory difficulties in term of old age [70]. Explaining giving up occupations as losing interest in them or being too old for them [98]. *Contributing to society* Trying to be satisfied or finding ways of being useful [37]. Being a mother continued to be important [48]. Use remaining abilities and contribute to the household [48]. Appreciating the value of still being able to do things and function [48]. Being engaged in volunteer work to feel useful and make a contribution to society [72]. Being someone to others, and oneself in a social context, by giving homemade items [98]. Contributing to the family [61]. Finding purpose in life by crochet baby clothes to donate to hospitals [90].	*Taking control and compensating* Mobilizing resources by accessing external services and friends and families [49]. Falling back on religion or life-values in times of stress as sources of comfort [47]. Doing life-long hobbies and habits to provide enjoyment and distraction from worries [52]. Using strategies to avoid mistakes due to memory loss [54]. Maintaining meaningful activities to cope with symptoms of dementia and the feeling of control [50]. Using old photographs and recounting stories to keep familiar ties and maintaining the experience in a socially coherent context [98]. Leisure activity counteract changes by keeping an active mind and give meaning in life [50]. Develop strategies to compensate for impairment [36]. Spending time and effort in planning and organising to meet difficulties better [36]. Writing notes to remember [40]. Using coping mechanisms trying to improve memory by cognitive exercise [47]. Using internet support groups to get knowledge of dementia and find ideas to manage changes [49]. Reduce household and external activities to manage stress [52]. Doing physical activity to delay deterioration [57]. Using external memory aid, structure of daily routine and medication to cope [70]. Using technology to provide “meaning and rescue” to each day [79]. Stopping certain occupations to avoid having accidents and misadventures [98]. Managing with assistance [57] Escaping emptiness and boredom with TV, creating the feeling that there is someone else at home [89]. Accessing groups with other people with dementia to be proactive in managing dementia [83]. Being proactive to contact home health care nurse, because she knew help will be needed [88]. Going to the gym as an active step to develop a social attribute, so that others do not define him excessively by his dementia [91]. Maintaining meaningful activities to cope with symptoms of dementia and the feeling of control [50]. Important with familiarity with place to perform activities independently [35]. Holding on to autonomy, defend themselves against the concerns of their partner and others [40]. Having alternative plans if something doesn’t go as planned [49]. Be in control of own situation by deciding who to inform about the diagnosis [80]. Continuing daily routine helps staying in control of the situation and their identity [93]. Not accepting being talked over or about, but taking steps to educate others about dementia [61]. Among some, music was an active, enriching and embedded part of their everyday lives and it enhanced their sense wellbeing [87]. Involvement in music gave people with dementia a degree of empowerment and control over their own lives [87]. *Reframing identity.* Redefining self [38]. Affirming one’s own identity and worth, by comparing with those who is less fortunate [37]. Comparing with others in same situation affirmed own approach to dementia [63]. Reoffering identity and place in the world, by deciding whether or not to accept help [48]. Making decisions help reaffirm a sense of self [48]. Taking responsibility for own personhood or relate closely to other members of the group of persons with dementia, in effort to bolster personhood [78]. Being a fighter [78]. Construct new self-narratives allowing to face the future with a certain equanimity [81]. Hope was balanced with the reality of a life well lived, and satisfaction of life, compared with those who was worse off [97]. Defining his role at a walking group to be a helper for others, whose needs are greater than his own [91]. Telling comprises a key element in the process of coming to terms with a diagnosis and co-constructing different sense of self [96].	*Position in life* Highlighting things one can still do [33]. Maintaining a positive view of oneself [33]. Experiencing hope by having a positive focus on life and abilities to change [50]. Looking forward, don’t look backward [43]. Positive attitudes towards their present and future [50]. Focusing on the present day and positive things in life [80]. Believing that it is not what happens to you that matters, it’s how you think about it [72]. Live in the moment, enjoy life [50]. Referring to dementia as a problem, not the end of the world. Don’t think about it [91]. Positive attitude give hope and counteract losses [50]. Focusing on remaining abilities [63]. Shift focus from dementia to living well [62]. Remaining positive and focus on the possibilities in life, rather than the losses [80]. Not letting dementia take over my life [68]. Putting some effort into it, and put some things behind you [75]. Planning ahead helped to accept the future with a sense of hope [50]. Hoping for the future, regardless the prospect [37]. Develop ways of managing the thoughts about the future/ Finding meaning in the future [38].	An active resistance to change by refusing to adapt or refusing to accept help [49]. Keep telling myself that none of this is important [39]. Trying not to think about moving to nursing home [41]. Resistance to make changes because it would mean acceptance of the progression of the decease [49]. Resisting change to fight stigma and threats of identity [49]. Avoid thinking of the future [55]. Taking no initiative [89]. Push dementia away [27]. Linguistic strategies to create emotional distance [27] Avoidance strategies like withdrawing and concealing difficulties [lead to isolation] [27]. Preventing themselves from thinking about the future [38]. Avoiding the influence of others who are further in the disease progression [72]. Holding on to the moment to protect themselves for the dreaded future [42]. Keeping occupied [71]. Being more active and do things as distraction [94]. Forget about the house and start a new life [in nursing home] [92]. Talking about others to get a distance between the dementia and oneself [47].

### Keep going and holding on to life as usual

This category relates to people with dementia continuing doing the same things they used to, taking 1 day at a time. They try to live their lives in the same way and hold on to the same activities as before they became ill. The underlying reason for this may be that they wish to do as much as they can before the progression of dementia reduces their abilities. The focus is on the present and to holding on to established routines and social relations. The category consists of the following subcategories: (a) *Preserving identity*; (b) *Normalising the situation*; and (c) *Contributing to society*.

*Preserving identity* refers to holding on to the identity that defines them as a person. They put effort into maintaining social roles and relations. They remind themselves and others of past achievements to preserve identity and self-esteem. By holding on to remaining aspects of themselves, they continue to live with the same sense of self as they have had for years.

*Normalising the situation* describes how people with dementia attempt to carry on with life and how they try to maintain a normal situation in daily life. It also refers to the effort they make to convince themselves and others that difficulties of memory loss or other challenges following dementia can be understood and explained as normal. They often compare themselves with others who forget things in order to normalise their own symptoms. By using this strategy, they seek to reduce their own worry and avoid negative response from surrounding people.

*Contributing to society* refers to the value of still being able to do meaningful activities and being useful. Many express a need or wish to use their remaining abilities and resources to help family or others for example by volunteer work.

### Adapting and adjusting to the demands from the situation

This category of coping describes how people adapt and adjust by changing their own expectations towards themselves and activities they can perform. They seek information about dementia to be able to plan and prepare for the future. It also involves being active and doing changes in order to handle the situation. The category consists of the subcategories: (a) *Taking control and compensating*; and (b) *Reframing identity.*

*Taking control and compensating* describes what the person does to continue being both physically and cognitively active. Additionally, in order to make life work despite their cognitive decline they find ways of doing things differently They emphasize that having regular routines, writing down important things and using technological aids are good tools to help them maintain function. The subcategory also relates to the willingness to ask for and accept practical help from others, decide who should know about the diagnosis, and stick to familiar routines and places.

*Reframing identity* refers to how the person build identity by thinking differently about themselves and comparing their lives to the lives of those who are worse off. They construct a new self-narrative as one more fortunate, which gives hope for the future and appreciation of life. Also, by being active in making decisions in life, they take responsibility for own personhood and autonomy.

### Accepting the situation

This category includes acknowledgement of the situation and the diagnosis of dementia. It is an acceptance of memory loss and other symptoms of dementia and a recognition of what they can do for themselves and when assistance from others is needed. The person with dementia reconciles with life as it is, though it can be a resigned acceptance. This category consists of the subcategory *position in life*.

*Position in life* is about acknowledging the dementia diagnosis and the consequences of the disease and focusing on strengths and possibilities. Dementia is described as a challenge or a problem, not “the end of the world”. Some have lived through challenging situations earlier in life, either it is related to health or e.g. having lived through a war. The positive orientation can be understood as a desire of living well, not letting dementia take over their life and having hopes for the future regardless the prospect.

### Avoiding the situation

People with dementia may experience challenging situations in which they feel unskilled or inadequate due to the cognitive decline. In addition, having a diagnosis of dementia and experience the condition develop can be stressful and threatening to the experience of security and sense of self. Coping by trying to avoid stressful situations could concern an *active resistance* to adaption, change or to accepting help because this may imply accepting dementia and the progression of the symptoms. The person with dementia can use strategies that actively redirects focus or refrain from the exposed situations. For instance, by changing subject in a conversation or avoiding situations by using distractions such as being active, keeping occupied or compensate. The focus is less on handling a “threat” that emerges, but more on keeping a distance and avoiding it in advance. This can include not thinking and talking about the future, withdrawal and not taking initiative because this could mean being exposed as cognitively impaired.

### Balancing the struggle of living with dementia

The four categories of strategies presented in the material do not necessarily follow each other in a linear process of coping ending in acceptance of the situation. Instead, they should be seen as potential strategies when meeting challenges and stress following dementia. The participants reported using several strategies at the same time and they employed different strategies depending on appraisals of the demands from the situation they encountered. Hence, the choice of strategies will be influenced by not only available resources of coping, but also the situation and context, and several strategies can be used for the same situation and challenge. The coping process and the four strategies described by people with dementia can be summed up in the overall theme *balancing the struggle of living with dementia*.

## Discussion

The main findings from this study revealed two main resources of coping: (1) Humour; and (2) Practical and emotional support, and four overall strategies in which people with dementia cope with the challenges they experience: (1) Keep going and holding on to life as usual; (2) Adapting and adjusting to the demands from the situation; (3) Accepting the situation; and (4) Avoiding the situation. The coping process and the four strategies can be summarized in the theme; balancing the struggle of living with dementia.

The two studies in our data material explicitly exploring coping in people with dementia had different perspective. Frazer et al. [47] studied how women who live alone with dementia cope in their everyday lives. They found that their participants were actively engaged in re-constructing their sense of self, using a variety of coping strategies. Sharp [18] described how people with dementia cope with the stress they experienced. The author highlighted the topic of learning to do things differently and establishing coping strategies that provide control. Her participants described individual potential for adapting and coping with the stressful aspects of living with dementia. Even though the two papers do not present their interview guides, there are reasons to believe that the participants have been asked to describe their ways of coping.

However, most of the papers included in our study did not ask the participants explicitly about their coping strategies, all articles described different ways in which people with dementia cope with challenges in their daily lives. Coping strategies are responses to stress such as specific challenges and daily struggles. Our findings describe both problem- and emotion- focused coping strategies. A problem-focused strategy can be to do something yourself, but it can also mean letting others do something for you when the aim is to minimize the stressful situation. Emotion-focused strategies may relieve a person’s situation by creating distance to the challenges, for instance by avoiding confrontations regarding symptoms and thereby shielding themselves from the stressful situation. The papers included in our study describe that people with dementia often use several strategies simultaneously. This is in line with the literature which report that more severe and acute stress situations demand the use of all available strategies and that people alternate between different strategies [11, 14–16]. Several papers convey how people with dementia keep going despite the rising challenges they meet due to progression of their cognitive and functional decline. In the early phase of the condition, many people have a strong will to continue living life as normal. This might be possible if one manages to mobilise available internal and external resources when approaching the specific situations [11]. Humour can be an effective coping strategy for making a threatening situation more harmless and thereby reducing stress. The experienced threats can vaporize if the person is able to make a joke and cover up the graveness of the situation. Through humour, the rational and sometimes harsh world can be kept stable as well as more tolerable [102]. Our study also showed that practical and emotional support are important coping resources for people with dementia. The findings are supported by a meta-synthesis of Eriksen et al. emphasising supportive interactions by family and friends as essential [23]. Supportive interactions enable people with dementia to prolong function and control in daily life, even though the areas of functioning gradually are becoming smaller. When symptoms increase and obstacles appear, our study gives the impression that most people with dementia fight the challenges. This is in line with a meta-synthesis conducted by Wolverson et al. [20] comprising the strengths that people can utilize in facing and fighting dementia, even leading to an experience of personal growth. Overall, fighting to keep going and overcome the daily struggles are signs of a problem-focused coping approach in people with dementia.

As the dementia condition progresses, and cognition as a personal coping resource declines, fighting the challenges will be more and more difficult. The person might experience that the ability to meet the situation changes and must therefore redefine what is important to him/her. Transformation in terms of new orientation and change of values (i.e. in relation to what is important in life), can be explained as response shift as described by Schwartz & Sprangers [103]. By «letting go» people with dementia can make a psychological shift from trying to change the unchangeable to acceptance and thereby deal with the decline [104]. People with dementia adapt by adjusting and compensating for their losses and challenges by selecting other goals or fewer domains in which to optimize their efforts and thereby sustain identity, function, and autonomy [105, 106]. In this process, emotion-focused strategies can help the person to adapt, reorient and regain balance. When balance is re-established, it may be relevant to use problem-focused strategies again.

Some of the studies refer to people with dementia expressing what they actually manage to do, despite their symptoms of decline. This can be seen as a particular way of understanding how values have been redefined. A recent meta synthesis showed that as the condition progresses, the lived space is narrowed for people with dementia. Førsund et al. [24] describe this process through the metaphor of the Russian “babushka doll,” which is a set of dolls of decreasing sizes that all fit inside one another one by one. Step- by- step, the person with dementia faces new challenges in how to cope.

People with dementia experience many challenges and a lot of stress. When life changes and the condition progresses, finding ways to cope is essential for making everyday life work and solve practical tasks. However, coping is also about finding ways to continue being oneself and to experience meaning and autonomy [11]. Thus, knowledge about coping resources and strategies in people with dementia is essential in dementia care. This insight is highly relevant to health care personnel whose role it is to help facilitate a supportive environment for the person with dementia. However, the information is also highly relevant for informal carers. They may support the person’s functioning by helping to facilitate and coordinate everyday living, but also by giving emotional and practical support. Though being able to do the things they still manage to do, perceptions of control continue to be a personal resource and may prevent experiences of helplessness and hopelessness, anxiety and depression [107].

### Limitations and strengths

We performed a systematic meta-synthesis and a transparent description of the selection process for the included articles have been presented. However, we acknowledge that a complete overview is not attainable, despite our approaches. The value of both individual reviewers and the use of pairs of researchers to evaluate the studies should be accredited.

The CASP criteria for qualitative studies consist of ten questions in which we have chosen to the first nine. These nine criteria assess the quality of the structure and the objective elements of the articles. The last and 10th question has not been considered as this is a subjective appraisal of the value of the research and the particular article as a whole. The10th question can be seen as important for the external validity of single study as a whole. This said, in our review we used parts of the articles describing the experience of coping and we re-analyzed and synthesized the material. A broader description of context could have strengthened the review.

Only nine of 74 studies included in this meta-synthesis had a longitudinal design. Therefore, it was not possible to describe if - or how - coping strategies change over time due to progression of the dementia. Further research is recommended to use longitudinal design and explore to what degree the patients at different stages of the dementia trajectory, use either problem-focused and/or emotional- focused coping strategies.

## Conclusions

People with dementia experience stress during the dementia trajectory and it is essential to find ways to cope in order to make everyday life work and to continue being oneself and experience meaning and autonomy. Thus, knowledge about coping resources and strategies in people with dementia is essential in dementia care. The insight is highly relevant to health care personnel and next of kin whose role it is to help facilitate a supportive environment for the person with dementia. This systematic meta-synthesis shows that people with dementia cope in different ways and use several parallel strategies in order to meet the challenges they face. The comprehensive meaning is understood as: *Balancing the struggle to live with dementia.*

Further research should reconsider using a longitudinal design and explore coping strategies in different stages of the dementia trajectory. It is also interesting to explore the influence of coping resources like humour and social and emotional support on coping strategies and capabilities of people with dementia. Last, but not least it is important to explore the success of coping strategies in order to support these strategies in people with dementia.

## Data Availability

All the articles included in this meta-synthesis are presented and accessible. The datasets used and analyzed during the current study are available from the corresponding author on reasonable request.

## Abbreviations

ASH: Anne Sofie Helvik
CASP: Critical appraisal skills programme
EKG: Ellen Karine Grov
EWT: Elisabeth Wiken Telenius
GHB: Guro Hanevold Bjørkløf
SE: Siren Eriksen
TIL: Tanja Louise Ibsen
